# Supplementary material for: Reconfigurable soft body trajectories using unidirectionally stretchable composite laminae
Source: Nat Commun. 2019 Aug 1;10:3464. doi: 10.1038/s41467-019-11294-7 (PMC6673692; doi:10.1038/s41467-019-11294-7)
Supplement: Supplementary file 1 — Supplementary information [file 41467_2019_11294_MOESM1_ESM.pdf]

Supplementary Information

Reconfigurable soft body trajectories using unidirectionally stretchable composite laminae

Sang Yup Kim[1†], Robert Baines[1†], Joran Booth[1], Nikolaos Vasios[2], Katia Bertoldi[2,3], Rebecca Kramer-Bottiglio[1*]

[1]Mechanical Engineering and Material Science, School of Engineering and Applied Science, Yale University, 9 Hillhouse Ave, New Haven, CT 06511, USA.

[2]School of Engineering and Applied Sciences, Harvard University, Cambridge, MA 02138, USA.

[3]Kavli Institute, Harvard University, Cambridge, MA 02138, USA.

Correspondence to: rebecca.kramer@yale.edu

Contents

Supplementary Movies 1 to 10 ………………………………… 2
Supplementary Methods ………………………………… 3
Supplementary Figures 1 to 5 ………………………………… 14
Supplementary Tables 1 and 2 ………………………..……..… 18

Supplementary Movies

Movie 1: Strain-limiting behavior of STAUD-prepreg

Movie 2: Contraction of pneumatic cylinder

Movie 3: Elongation of pneumatic cylinder

Movie 4: Rotation of pneumatic cylinder

Movie 5: Thin planar actuator bending

Movie 6: Bending of pneumatic cylinder

Movie 7: Octopus-like tentacle movement with localized patches

Movie 8: Planar octopus-inspired actuator

Movie 9: Balloon trajectory programming

Movie 10: Membrane-actuated tensegrity

Supplementary Methods

We built a fiber-winding machine to fabricate STAUD-prepreg with tunable properties in a scalable fashion. The fiber winder consists of a spool holster, a linearly-translating fiber distributor, a rotating winding mandrel. The fiber distributor was prepared using a ball screw (Model #: MTF06011, THK) attached to a stepper motor (Product #: 324, Adafruit) and a metal eye through which the fiber travels. The winding mandrel is an aluminum drum of 175 mm (ϕ) × 225 mm (h) attached to another stepper motor. The metal eye holds the fiber in constant tension as it spools from its holster onto the drum. Both motors are controlled via an Arduino with an attached Adafruit V2 motor shield. Precise inter-fiber spacing of composite lamina is accomplished by varying the rotation rate of the shaft and drum relative to one another.

To conduct uniaxial pull-to-failure tension tests, we fabricated rectangular laminae samples with three distinct inter-fiber spacings, (ℓ = 1, 2, and 6 mm), as well as three fiber angles, ($\theta = 0°$, $45°$, and $90°$), by laser-cutting the bulk laminae into discrete units (VLS 3.50, Universal Laser Systems). 12.7 mm-long tabs served as grip interfaces on the samples. The width of the tabs on a particular sample matched the sample's width w. Lengths, L (the distance between the tabs), across all angle/spacing combinations remained fixed at 101.6 mm. Sample widths for the $\theta = 45°$ and $\theta = 0°$ cases were 12.7 mm, but for $\theta = 90°$, was 25.4 mm. Sample average thicknesses, t, were individually characterized using a Zeiss Smartzoom 5 digital microscope. Later, we consider individual thicknesses to calculate stress-strain curves for each sample.

We performed unidirectional quasi-static tensile tests using a servo-hydraulic test machine (Model 3345, Instron). We considered a total of forty-five samples—five of each fiber orientation/spacing combination—to account for inter-sample variance. Pneumatic grips set to 40 psi provided sufficient force to hold $0°$ samples without allowing slip. Standard set-screw grippers were used for the $90°$ and $45°$ samples. We marked test samples with equidistant fiducials (Product#: 846, MG chemicals) about the midpoint of their longitudinal axes, and extracted true strains by tracking the fiducials in high-definition camera footage taken during each trial. For the $90°$ and $45°$ lamina, uniaxial tensile loading was applied at 50 mm/min until failure. In the $0°$ case, we slowed the loading speed to 25 mm/min.

Stress is found by dividing the pull force by the cross-sectional area of the midpoint of the sample:

$$\sigma = \frac{F}{A} \tag{1}$$

To account for changing cross sectional area of the samples we apply a differential form of Poisson's law.

$$\begin{aligned} A &= tw\left(1 - 2\delta + \delta^2\right) \\ \delta &= 1 - \left(1 + \frac{\Delta L}{L}\right)^{-\upsilon} \end{aligned} \tag{2}$$

Here, d is the induced contraction normal to the direction of applied strain. Thus, we calculate true stress and observe true strain.

Experimental stress strain curves are summarized in Supplementary Figure 1c, and expanded in more detail in Fig. 2a of the main manuscript. Initial Young's moduli for lamina principal directions 1 and 2, as well shear direction 6 (Supplementary Figure 1), were calculated via averaged linear fit of sample the stress-strain curves from 0.02-0.03 strain (linear region). Off-axis tensile testing of a $\theta = 45°$ sample, as recommended in previous work [1], allowed us to determine the shear modulus of the lamina. Applying a rotation to the tensile stress, σ_x, to transform it about the material axes and obtain laminate coordinate shear stress is simply:

$$\sigma_6 = -\sigma_x \sin\theta \cos\theta \quad (3)$$

where $\theta = -45°$. We acquired shear modulus, G_6, in a similar fashion as the principal lamina moduli, but instead of purely longitudinal strain, we considered the difference between longitudinal and transverse strain (which is in-plane shear strain for 45° off-axis case) [2]. We compare experimentally calculated moduli to theoretical moduli arising from the rule of mixtures (4) and inverse rule of mixtures (5), (6), respectively [2]. We assume the fibers and matrix are each isotropic.

$$E_1 = V_f E_f + V_m E_m \quad (4)$$

$$\frac{1}{E_2} = \frac{V_f}{E_f} + \frac{V_m}{E_m} \quad (5)$$

$$\frac{1}{G_6} = \frac{V_f}{G_f} + \frac{V_m}{G_m} \quad (6)$$

Here, E_1 and E_2 are the elastic moduli of the composite in the 1 or 2 lamina coordinate system directions, respectively, E_f is the elastic modulus of the fibers, G_f is the shear modulus of the fibers, E_m is the elastic modulus of the matrix, G_m is the shear modulus of the matrix, V_m is the volume fraction of the matrix, and V_f is the volume fraction of fibers. We used 68 kPa for the elastic modulus of the matrix material. This value was as determined through tensile tests of a pure Ecoflex 030 sample.

Spun polyester yarn fiber has a range of reported elastic moduli depending on degree of twist [3], tensile conditions during integration to another structure (in our case, spooling on drum for composite manufacture) [4], and a host of other factors, including temperature and humidity [5]. In fact, twisting of individual filaments into bundle can decrease Young's modulus by nearly 33 % [5]. We opted to use a modulus of 1.3 GPa, as discerned from previous work [6]. Following the assumption of fiber isotropy, the shear modulus for the polyester fibers was assumed to be 1.3

GPa as well. The shear modulus of Ecoflex 030, by assuming incompressibility and evoking the isotropic material relation is given as:

$$G = \frac{E}{2(1+\upsilon)} \tag{7}$$

was found to be 22 kPa. This shear modulus is consistent with previously reported values [7].

Theoretically and experimentally calculated initial elastic moduli lie in Supplementary Table S1. From the table, it is clear that a larger volume fraction of fibers, corresponding to a smaller inter-fiber spacing, ℓ, make a lamina stiffer. We see this trend for both 1 and 2 material axes. Expectantly, E_1 is greater than E_2, because the fibers dominate laminae mechanical responses at $\theta = 0^{\circ}$.

The rule of mixtures tends to overestimate elastic modulus, as it does not take into account micro-voids and geometrical imperfections present in real material samples. This fact helps explain why theoretical elastic moduli values are for the most part above the experimental ones reported above. Imperfect sample alignment during testing could have also contributed to the discrepancy between experimental and theoretical values. The mechanical properties of highly anisotropic composites are very sensitive to fiber alignment, and slightly offset angles can impact behavior substantially [5]. Perhaps this is why we see higher experimental in-plane shear moduli.

Laminae lengths and widths are much greater than their respective thicknesses, so we abide by the assumption posited for in-plane stress-strain relations for thin laminae. Traditionally, the stiffness or compliance matrix constructed for laminae uses fixed moduli and consequently is a linear mapping between strain and stress [2]. We account for the non-linear material behavior of the lamina under plane stress, in addition to the geometric non-linearity of the lamina in its deformed state through a moving Eulerian coordinate system in the fashion of Chou [5], based on work by Hahn and Tsai [8]. Neglecting shear-coupling terms to simplify the expression, the stress-energy per unit area of a deformed lamina can be written as:

$$\begin{aligned} W^* = {} & \frac{1}{2}S_{11}\sigma_1{}^2 + \frac{1}{3}S_{111}\sigma_1{}^3 + \frac{1}{4}S_{1111}\sigma_1{}^4 + \\ & S_{12}\sigma_1\sigma_2 + \frac{1}{2}S_{22}\sigma_2{}^2 + \frac{1}{3}S_{222}\sigma_2{}^3 + \frac{1}{4}S_{2222}\sigma_2{}^4 + \\ & \frac{1}{2}S_{66}\sigma_6{}^2 + \frac{1}{4}S_{6666}\sigma_6{}^4 \end{aligned} \tag{8}$$

Here, S_{ij}, S_{ijk}, and S_{ijkl} are compliance terms. Chou constructs the simplified compliance matrix following the derivative of the complementary strain energy function

$$\delta W^* = e_{ij}\delta\sigma_{ij} \tag{9}$$

To obtain:

$$
\begin{aligned}
e_1 &= S_{11}\sigma_1 + S_{111}{\sigma_1}^2 + S_{1111}{\sigma_1}^3 + S_{12}\sigma_2 \\
e_2 &= S_{22}\sigma_2 + S_{222}{\sigma_2}^2 + S_{2222}{\sigma_1}^3 + S_{12}\sigma_1 \\
e_6 &= S_{66}\sigma_6 + S_{6666}{\sigma_6}^3
\end{aligned} \tag{10}
$$

Note the e and σ are Eulerian terms referring to the current principal material coordinates. We can relate engineering strain ε to Eulerian strain e with the following relation [9]:

$$
\varepsilon = \left(\frac{1}{1-2e} \right)^{-1/2} - 1 \tag{11}
$$

The simplified compliance matrix which relates in-plane stresses to in-plane strains along the principal material axes 1 and 2 and shear direction 6 is constructed as such:

$$
\mathbf{S} = \begin{bmatrix} S_{11} + S_{111}\sigma_1 + S_{1111}{\sigma_1}^2 & S_{12} & 0 \\ S_{12} & S_{22} + S_{222}\sigma_2 + S_{2222}{\sigma_1}^2 & 0 \\ 0 & 0 & S_{66} + S_{6666}{\sigma_6}^2 \end{bmatrix} \tag{12}
$$

Constants S_{11}, S_{22}, S_{12}, and S_{66} equal $\frac{1}{E_1}$, $\frac{1}{E_2}$, $\frac{-\upsilon_{12}}{E_1}$, and $\frac{1}{G_6}$, respectively, and were obtained based on initial experimental moduli tabulated in Supplementary Table S1. Higher order terms S_{ijk} and S_{ijkl} were determined by fitting experimental stress strain curves according to steps outlined in previous work [5]. MATLAB least squares functions were used for the fitting. Off-axis laminae may be rotated about principal axes to a local loading orientation θ by applying a transformation matrix.

$$
\mathbf{T} = \begin{bmatrix} \cos^2\theta & \sin^2\theta & -2\sin\theta\cos\theta \\ \sin^2\theta & \cos^2\theta & 2\sin\theta\cos\theta \\ -2\sin\theta\cos\theta & 2\sin\theta\cos\theta & \cos^2\theta - \sin^2\theta \end{bmatrix} \tag{13}
$$

The stress-strain relations for a lamina can now be written as:

$$
[\mathbf{e}] = [\mathbf{T}]^{-1}[\mathbf{S}][\mathbf{T}][\boldsymbol{\sigma}] \tag{14}
$$

To account for strain relaxation of elastomers as a result of cyclic loading, also known as the Mullins effect [10], we adjust predicted strains arising from our analytical model. A polynomial damage function was constructed based on the stress-strain behavior of the fibrous composite that was un-stretched, and then stretched up to 70% strain 10 times in succession. Tests were conducted up to 20 cycles, and it was determined that there is negligible (< 5 %) stress-strain change after the 10 cycles threshold. This finding is consistent with other work [10-12]. The damage function relates an un-stretched strain to a cycled strain at the same stress value. We chose G_c to be a cycle-depending scaling factor such that for cycle c:

$$\Gamma_c = \begin{cases} 1 & \text{if} \quad c = 1 \\ \dfrac{\beta_0 + \beta_1\sigma + \beta_1\sigma^2 + \beta_1\sigma^3}{\beta_0' + \beta_1'\sigma + \beta_1'\sigma^2 + \beta_1'\sigma^3} & \text{if} \quad c > 1 \end{cases} \tag{15}$$

Here, b_i are coefficients for the third-order polynomial fit of the average un-cycled curve, while b¢are those for the average cycled curve. We can write our final formulation for predicting lamina strain as a function of input stress and cycle number:

$$[\mathbf{e}] = \Gamma_c [\mathbf{T}]^{-1}[\mathbf{S}][\mathbf{T}][\boldsymbol{\sigma}] \tag{16}$$

For n layered-laminate, where each layer k has thickness $z_k - z_{k-1}$ and its mid-plane is a distance z_k from the mid-plane of the total laminate [2]:

$$\sum_{k=1}^{n} \int_{z_{k-1}}^{z_k} [\boldsymbol{\sigma}]_{x,y}^{k} \, dz = \sum_{k=1}^{n} \int_{z_{k-1}}^{z_k} [\mathbf{Q}]_{x,y}^{k} [\boldsymbol{\varepsilon}]_{x,y}^{k} \, dz \tag{17}$$

Q is the stiffness matrix. As we are interested in the continuous strains through the thickness of the laminate, we must consider the inversion of the above relation. Note that:

$$[\mathbf{Q}] = [\mathbf{S}]^{-1} \tag{18}$$

Having accounted for material non-linearity (not present in assumptions in classical laminate theory) according to third order polynomial modeling based off the complementary stress energy density function, geometric non linearity with an Eulerian coordinate system, as well as cyclic stress-strain behavior of our matrix material with a Mullins damage function, we can model the creation of STAUD-prepreg laminates. We dub this adaptation Augmented Classical laminate theory (ACLT). We express it mathematically as:

$$\sum_{k=1}^{n} \int_{z_{k-1}}^{z_k} [\mathbf{e}]_{x,y}^{k} \, dz = \Gamma_c \left[\sum_{k=1}^{n} [\mathbf{S}]_{x,y}^{k \;\; -1} \right]^{-1} \sum_{k=1}^{n} \int_{z_{k-1}}^{z_k} [\boldsymbol{\sigma}]_{x,y}^{k} \, dz \tag{19}$$

Using our adapted formulation of classical laminate theory, we predicted the engineering strain of cylindrical actuators as a function of applied input stress. We conducted tests on three baseline cases of actuators (n = 1): 90°, 0°, and 45° fiber orientation. We also explored creation of laminate actuators (n = 2): 90°+0° and 90°+45°. We cycled the cylinders 10 times before conducting each test, breaking them in to a repeatable range. We adjusted analytical results with the strain relaxation damage function detailed above. We acquired stress by transforming input pressure read from a pressure sensor (Model#: 015PGAA5, Honeywell) using thin-walled pressure vessel equations.

Our model assumes gravitational forces acting on the cylinder are negligible. We ignore inter-layer adhesive layer thicknesses as well. We model an inflating pneumatic cylinder as a thin

wall pressure vessel (Supplementary Figure 2) because its wall thickness is much smaller than its radius [13]. Circumferential hoop and axial stresses are the only forces acting on a thin-walled pressure vessel and are purely planar forces that give a mapping between applied pressure and stresses experienced in the laminate coordinates.

$$\sigma_{axial} = \frac{pr}{2t} \qquad \sigma_{hoop} = \frac{pr}{t} \tag{20}$$

where p is the known input pressure, r is the radius of the cylinder, and t is the thickness of the wall.

In the 0° cylindrical actuator case, inflation causes transverse expansion, which subsequently induces contraction as fibers pull the bottom of the actuator up. We modeled the fiber deformation to have constant curvature, and applied the geometrical constraints detailed in Supplementary Figure 3 to calculate theoretical contraction based on theoretical transverse strain. In Supplementary Figure 3, l is the resulting length of the cylinder after it has contracted some distance Δl from an original length l_0. The induced arc of deformation is approximated by a secant line of length $\frac{l_0}{2}$, as pictured. The instantaneous radius, r, is updated based on the theoretical transverse strain: $r = \varepsilon r_0 + r_0$. Note the relation depicted by Supplementary Figure 3 holds only when

$$\frac{l_0}{2} > (r - r_0) \tag{21}$$

Finally, we must consider the thickness change of the vessel walls as a function of expansion to get an accurate representation of the stress states. In line with our planar stress assumption, out-of-plane stresses are not considered, and we can express the instantaneous thickness of the pneumatic actuator as

$$t = t_0 \varepsilon_z + t_0 \tag{22}$$

Since

$$\varepsilon_z = -\upsilon \varepsilon_x - \upsilon \varepsilon_y \tag{23}$$

Fig. 2a and 3a in the main manuscript show good agreement between experimentally observed and analytically predicted deformation of the various lamina-wrapped cylindrical actuators. For the 0° case, the model reliably predicts up to a certain value of strain, usually around 20 %, after which geometric effects not considered by our model, including fiber anchoring to the end caps of the inflating cylinder, begin to govern the stiffness of the system so we see significant deviation. Additional differences between theoretical and experimental strain values may have

arisen since we inflated the cylinders at a greater rate (× 20) than quasi-static conditions on of which the material moduli were derived. As such, our experimental curves may reflect hyper-viscoelastic effects. Overall, we achieve reliable prediction of deformation of soft bodies clad in sequences of angle-ply laminates in a range of strains suited to soft robotic applications.

In an effort to validate our experimental and analytical findings using ACLT but also to enable the modelling of more complex geometries and multi-layered STAUD-prepeg laminate actuators, we performed finite element analysis (FEA) simulations using Abaqus (SIMULIA, Providence RI) commercial software. FEA allowed us to fully resolve the strain and stress distributions of each laminae and further account for boundary effects in the various cylindrically shaped actuators. All models were generated using shell elements (S4R) using the composite shell section formulation that enables numerical integration of composite shells consisting of several laminae in various orientations. We performed nonlinear quasi-static analyses using Abaqus/Explicit to simulate the deformation of cylindrically shaped actuators upon inflation. To ensure quasi-static conditions in the simulations, we verified that the kinetic energy associated with the finite loading and deformation rate of the actuators was at all times negligible compared to the strain energy in each model. All cylindrically shaped actuators were assumed to have strain-free caps, one of which was held fixed to eliminate rigid body translations and rotations. The actuators were inflated using the fluid-filled cavity interaction, corresponding to volume-controlled loading conditions. Each STAUD-prepreg lamina was modelled as an anisotropic continuum 3D shell whose constitutive behavior is governed by the Holzapfel-Gasser-Ogden (HGO) anisotropic hyper-elastic continuum model [14]. The strain energy density function associated with the HGO model takes the form,

$$\mathcal{W} = C_{10}(I_1 - 3) + \frac{k_1}{2k_2}\left(e^{k_2(I_4-1)^2} - 1\right) \tag{24}$$

where,

$$I_1 = \mathrm{tr}[B] \tag{25}$$

$$I_4 = (F \cdot \mathbf{M}) \cdot (F \cdot \mathbf{M}) \tag{26}$$

and $B = F \cdot F^{\mathrm{T}}$ is the left Cauchy-Green deformation tensor and M is the unit vector along the fiber direction in the reference configuration. The material parameters of the constitutive model, namely C_{10}, k_1, and k_2 are determined by fitting the model to experimental data, preferably containing information for the behavior of the sample in various fiber orientations. Here, we used nominal stress-nominal strain data corresponding to uniaxial extension experiments along 0°, 45°, and 90° orientations, and we performed non-linear least square fits against the corresponding stress-strain expressions for the HGO model. In the following section, we determine the nominal stress-strain relationships for the uniaxial extension of a thin film obeying the HGO model.

The nominal stress tensor can be determined from the strain energy function,

$$S = \frac{\partial \mathcal{W}}{\partial F} = \det F \cdot F^{-1} \cdot \sigma \tag{27}$$

where σ is the Cauchy stress tensor, which from an HGO anisotropic solid takes the form,

$$\sigma = -pI + 2\frac{\partial W}{\partial I_1} F \cdot F^T + 2\frac{\partial W}{\partial I_4} m \otimes m \tag{28}$$

where p is a hydrostatic stress and $m = F \cdot M$ is the unit vector along the fiber direction in the current (deformed) configuration. The deformation gradient tensor for an incompressible HGO anisotropic hyper-elastic material subjected to uniaxial extension has the form,

$$F = \lambda_x e_1 e_1 + \lambda e_2 \boldsymbol{e_2} + \lambda_z e_3 \boldsymbol{e_3} \tag{29}$$

and incompressibility implies that,

$$\boldsymbol{\lambda_x = \frac{1}{\lambda\lambda_z}} \tag{30}$$

The corresponding left Cauchy-Green deformation tensor $\mathbf{B} = \mathbf{F} \cdot \mathbf{F}^{\mathrm{T}}$ follows,

$$\mathbf{B} = \frac{\mathbf{1}}{\boldsymbol{\lambda^2\lambda_z^2}} e_1 e_1 + \boldsymbol{\lambda^2 e_2 e_2} + \boldsymbol{\lambda_z^2 e_3 e_3} \tag{31}$$

Given that the loading (extension) direction is along the $\boldsymbol{e_2}$ unit vector, we can further define θ

as the angle between the fiber direction $\boldsymbol{s}$ and the loading direction. The fiber direction in the reference configuration can thus be expressed as

$$\mathbf{M} = \boldsymbol{sin\,\theta\, e_1 + cos\,\theta\, e_2} \tag{32}$$

The fourth pseudo-invariant I_4 of the left Cauchy-Green tensor B is given by,

$$I_4 = (F \cdot \mathbf{M}) \cdot (F \cdot \mathbf{M}) = \lambda^{-2}\lambda_z^{-2}\, sin^2\,\theta + \lambda^2 \cos^2\theta \tag{33}$$

Furthermore, we have that,

$$\frac{\partial W}{\partial I_1} = C_{10}$$

$$\frac{\partial W}{\partial I_4} = k_1(I_4 - 1)e^{k_2(I_4-1)^2} \tag{34}$$

$$m = F \cdot \mathbf{M} = \lambda^{-1}\lambda_z^{-1}\, sin\,\theta\, e_1 + \lambda\, cos\,\theta\, e_2$$

$$m \otimes m = \lambda^{-2}\lambda_z^{-2} e_1 e_1 + \lambda_z^{-1}\, sin\,\theta\, cos\,\theta\,(e_1 e_2 + e_2 e_1) + \lambda^2\, cos^2\,\theta\, e_2 e_2$$

Therefore, combining the expressions above, the components of the Cauchy stress tensor σ_{ij} may be calculated as follows,

$$\sigma_{11} = -p + 2C_{10}\lambda^{-2}\lambda_z^{-2} + 2k_1\lambda^{-2}\lambda_z^{-2}\, sin^2\,\theta\,(I_4 - 1)e^{k_2(I_4-1)^2}$$

$$\sigma_{12} = 2k_1\lambda_z^{-1}\, sin\,\theta\, cos\,\theta\,(I_4 - 1)\, e^{k_2(I_4-1)^2}$$

$$\sigma_{13} = 0$$

$$\sigma_{21} = 2k_1\lambda_z^{-1}\, sin\,\theta\, cos\,\theta\,(I_4 - 1)\, e^{k_2(I_4-1)^2} \tag{35}$$

$$\sigma_{22} = -p + 2C_{10}\lambda^2 + 2k_1\lambda^2\, cos^2\,\theta\,(I_4 - 1)e^{k_2(I_4-1)^2}$$

$$\sigma_{23} = 0$$

$$\sigma_{31} = 0$$

$$\sigma_{32} = 0$$

$$\sigma_{33} = -p + 2C_{10}\lambda_z^2$$

Finally, the nominal stress tensor components are determined as

$$S_{11} = F_{11}^{-1}\sigma_{11} = -p\lambda\lambda_z + 2C_{10}\lambda^{-1}\lambda_z^{-1} + 2k_1\lambda^{-1}\lambda_z^{-1} \, sin^2\theta\,(I_4 - 1)e^{k_2(I_4-1)^2}$$

$$S_{12} = F_{11}^{-1}\sigma_{12} = 2k_1\lambda\, sin\,\theta\, cos\,\theta\,(I_4 - 1)\, e^{k_2(I_4-1)^2}$$

$$S_{13} = F_{11}^{-1}\sigma_{13} = 0$$

$$S_{21} = F_{22}^{-1}\sigma_{21} = 2k_1\lambda^{-1}\lambda_z^{-1}\, sin\,\theta\, cos\,\theta\,(I_4 - 1)\, e^{k_2(I_4-1)^2}$$

$$S_{22} = F_{22}^{-1}\sigma_{22} = -p\lambda^{-1} + 2C_{10}\lambda + 2k_1\lambda\, cos^2\theta\,(I_4 - 1)e^{k_2(I_4-1)^2} \quad (36)$$

$$S_{23} = F_{22}^{-1}\sigma_{23} = 0$$

$$S_{31} = F_{33}^{-1}\sigma_{31} = 0$$

$$S_{32} = F_{33}^{-1}\sigma_{32} = 0$$

$$S_{33} = F_{33}^{-1}\sigma_{33} = -p\lambda_z^{-1} + 2C_{10}\lambda_z$$

Since the sample is extended along e_2, the traction free conditions on faces e_1 and e_3 suggest that

$$S_{11} = 0 \;\Rightarrow -p\lambda\lambda_z + 2C_{10}\lambda^{-1}\lambda_z^{-1} + 2k_1\lambda^{-1}\lambda_z^{-1}\, sin^2\theta\,(I_4 - 1)e^{k_2(I_4-1)^2} \;=\; 0 \quad (37)$$

$$S_{33} = 0 \Rightarrow -p\lambda_z^{-1} + 2C_{10}\lambda_z = 0 \Rightarrow p = 2C_{10}\lambda_z^2 \quad (38)$$

Combining the last two equations, leads to the following non-linear equation

$$\lambda_z^4 = \lambda^{-2}\left[1 + \frac{k_1}{C_{10}} sin^2\theta\,(I_4(\lambda_z) - 1)e^{k_2(I_4(\lambda_z)-1)^2}\right] \quad (39)$$

where

$$I_4(\lambda_z) = \lambda^{-2}\lambda_z^{-2}\, sin^2\theta + \lambda^2\cos^2\theta \quad (40)$$

The non-linear equation needs to be solved with respect to λ_z for each value of λ. Once λ_z is known, the nominal stress S_{22} along the extension direction can be determined using,

$$S_{22} = 2C_{10}(\lambda - \lambda_z^2\lambda^{-1}) + 2k_1\lambda\, cos^2\theta\,(I_4 - 1)e^{k_2(I_4-1)^2} \quad (41)$$

Having determined an expression for the nominal stress component along the uniaxial extension direction, we fit the analytical expression to experimental data of uniaxial extension of samples at $\theta = 0°, 45°$ and 90°. We did this for each STAUD-prepreg inter-fiber spacing. The fitted material properties for each inter-fiber spacing are summarized in Supplementary Table S2.

Supplementary References

[1] M.-J. Pindera and C. T. Herakovich, Shear characterization of unidirectional composites with the off-axis tension test. *Experimental Mechanics*, **26**, 103–112 (1986).

[2] I. M. Daniel, O. Ishai, *Engineering mechanics of composite materials* (Oxford university press, New York, ed. 2, 2006).

[3] S. Rajendran, S. Ramasamy, and S. P. Mishra, Tensile behavior of polyester yarns modified by trichloroacetic acid–methylene chloride treatment. *Journal of Applied Polymer Science*, **59**, 99–108 (1996).

[4] V. Midha, A. Mukhopadhyay, R. Chattopadhyay, and V. K. Kothari, Studies on the Changes in Tensile Properties of Sewing Thread at Different Sewing Stages. *Textile Research Journal*, **79**, 1155–1167 (2009).

[5] Chou, T-W., Microstructural Design of Fiber Composites (Cambridge University Press, Cambridge, 1992).

[6] M. Zubair, thesis, Technical University of Liberec (2017).

[7] J. L. Sparks et al., Use of silicone materials to simulate tissue biomechanics as related to deep tissue injury. Adv Skin Wound Care, **28**, 59–68 (2015).

[8] H. T. Hahn, S. W. Tsai, Nonlinear elastic behavior of unidirectional composite laminae. *J. Compos. Mater.*, **7**, 102–118 (1973).

[9] S. Luo and T. Chou. Finite Deformation and Nonlinear Elastic Behavior of Flexible Composites. *Journal of Applied Mechanics*, **55** (1988).

[10] J. Diani, B. Fayolle, and P. Gilormini, A review on the Mullins effect. *European Polymer Journal*, **45** (2009).

[11] J. C. Case, E. L. White, and R. K. Kramer, Soft Material Characterization for Robotic Applications. *Soft Robotics*, **2** (2015).

[12] A. Dorfmann and R.W. Ogden, A constitutive model for the Mullins effect with permanent set in particle-reinforced rubber. *International Journal of Solids and Structures*, **41** (2004).

[13] Roy R. Craig, Jr, *Mechanics of Materials.* (John Wiley & Sons, Hoboken, NJ, ed. 3, 2011).

[14] A. Holzapfel, T. C. Gasser, and R. W. Ogden. A new constitutive framework for arterial wall mechanics, *J Elast.*, **61**:1-48 (2000).

Supplementary Figures

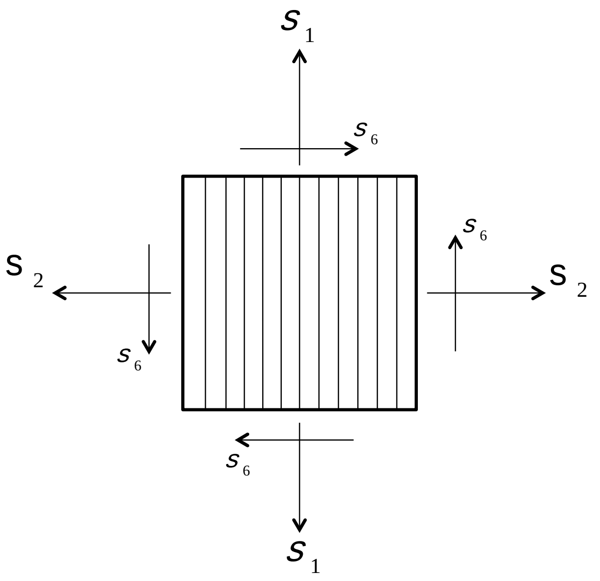

Supplementary Figure 1. Schematic defining lamina principal directions. (1) represents stresses and strains in the direction of embedded fibers, while (2) is transverse to the direction of the fibers. (6) is the in-plane shear component.

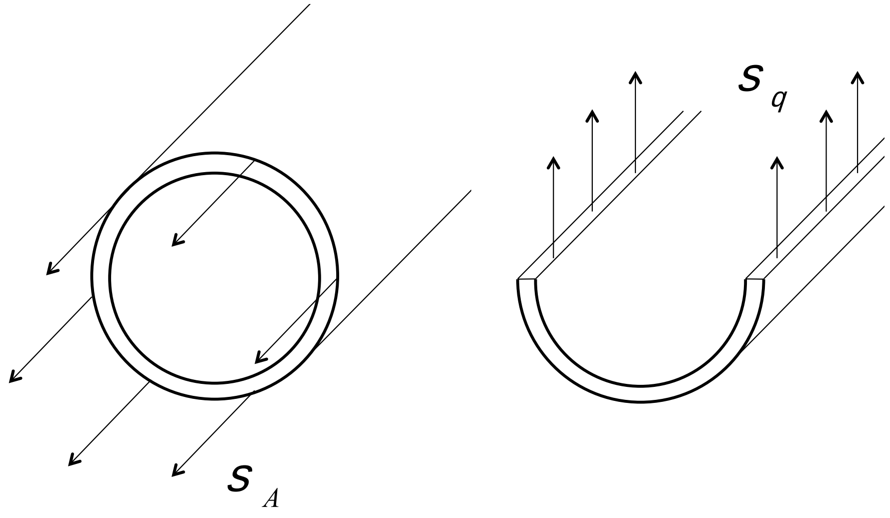

Supplementary Figure 2. Cross section a of thin-wall cylindrical pressure vessel illustrating the distribution of planar stresses. We model our pneumatic actuators under this assumption. Left: tangential hoop stress from cutting the cylinder along its height. Right: axial planar stress observed by cutting the cylinder through its width.

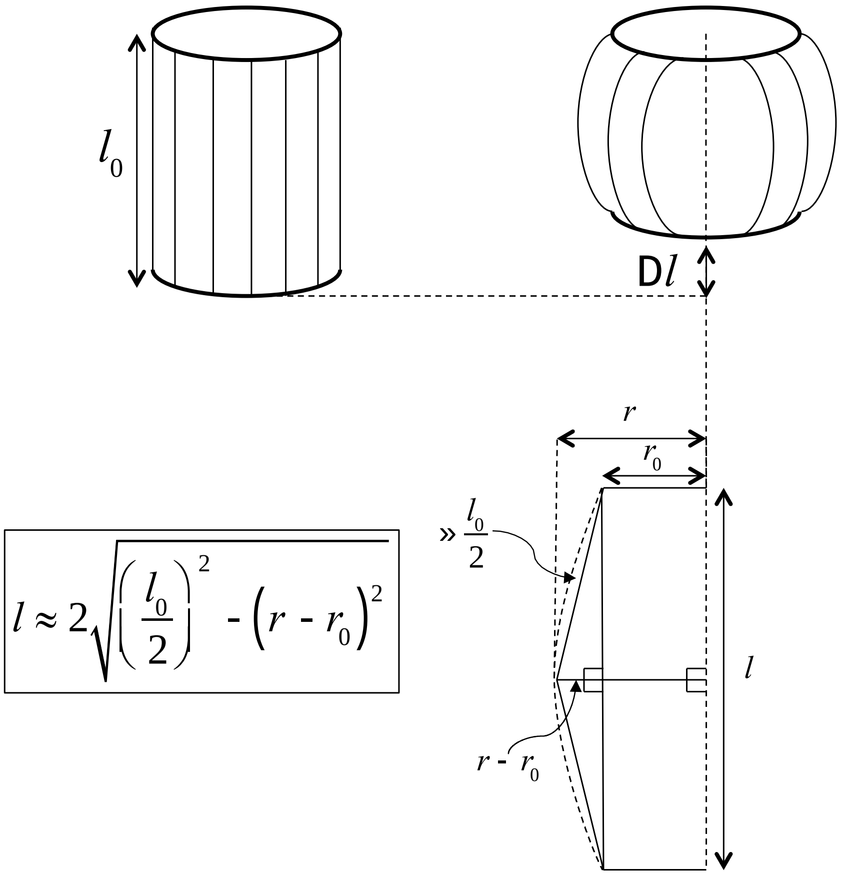

Supplementary Figure 3. Geometric model to account for the contraction of cylindrical pneumatic actuators wrapped with 0° STAUD-prepreg. When inflating, an initial radius r_0 increases to r. Meanwhile, an initial length l_0 shortens by Δl due to fibers "pulling up" the bottom of the cylinder. Approximating the deformed arc as two equivalent right triangles, the induced arc of deformation can be written as $\frac{l_0}{2}$ by the Pythagorean theorem.

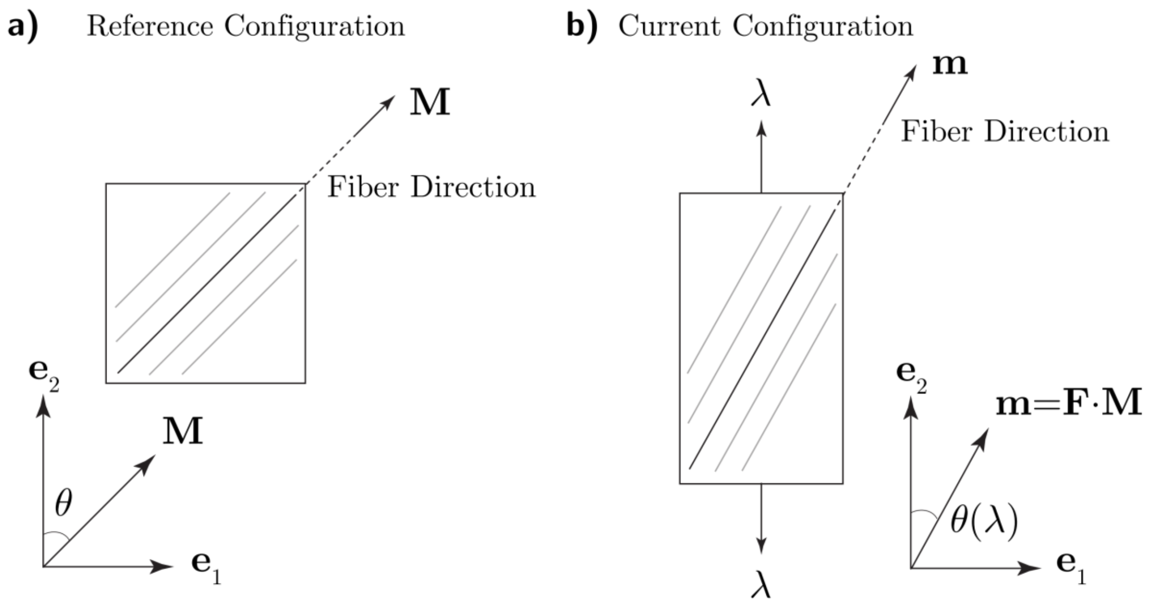

Supplementary Figure 4. Schematic of a fiber-reinforced hyper-elastic material subjected to uniaxial extension. a) $\mathbf{M}$ is the unit vector along the fiber direction in the reference configuration and $\boldsymbol{\theta}$ is the angle between the fiber and loading directions. b) $\boldsymbol{m}$ is the unit vector along the fiber direction in the current configuration after applying a uniaxial stretch equal to $\boldsymbol{\lambda}$.

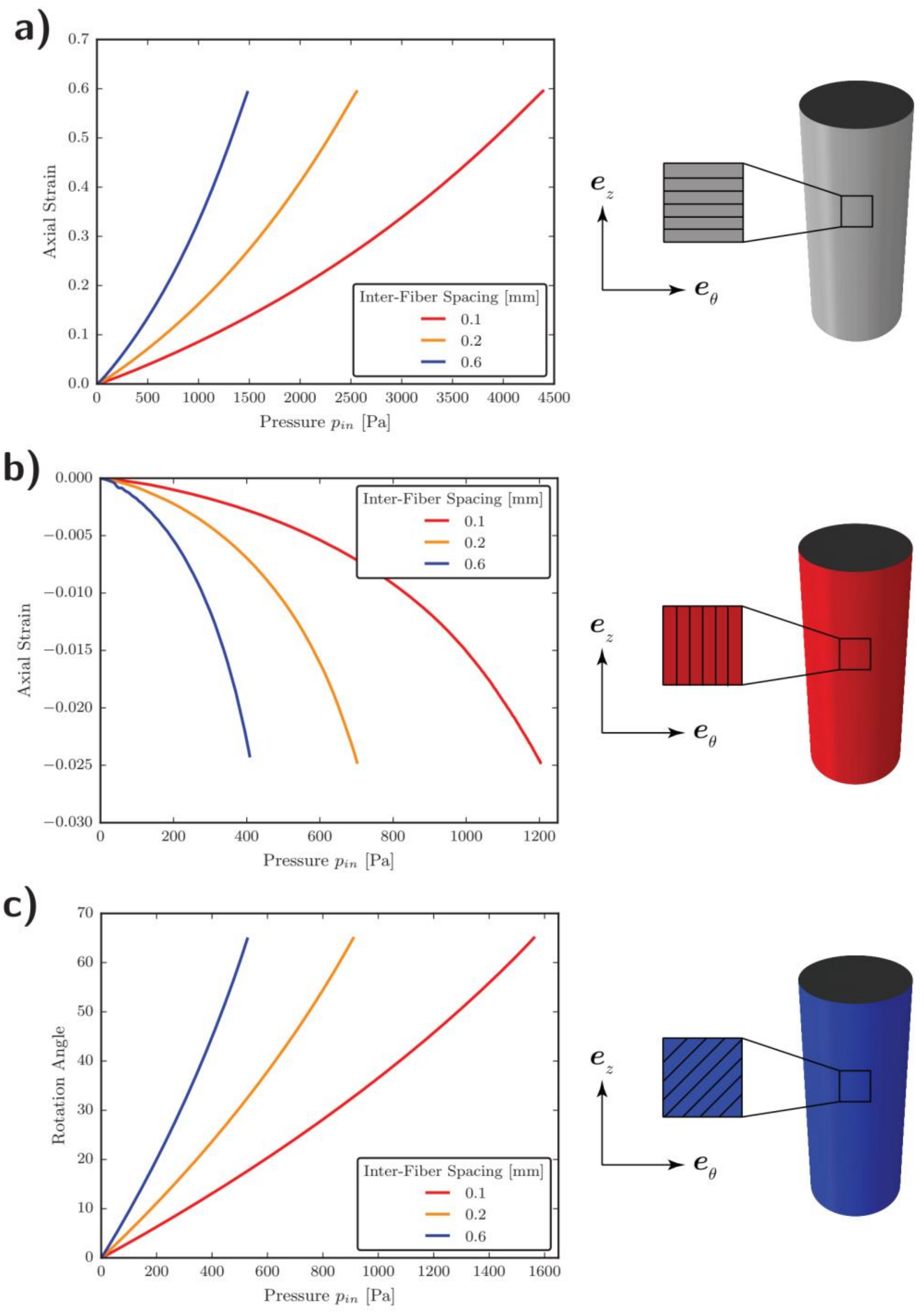

Supplementary Figure 5. FEA results for the quasi-static inflation of cylindrical actuators consisting of a single STAUD-prepreg, with three different inter-fiber spacings, wrapped at three different orientations. a) STAUD-prepreg wrapped at 90° produces an actuator that extends

upon inflation. b) STAUD-prepreg wrapped at 0° elicits an actuator that contracts upon inflation. c) STAUD-prepreg wrapped at 45° creates an actuator that twists upon inflation.

Supplementary Tables

Experimental versus Theoretical Moduli			
Inter-Fiber Spacing [mm]	Directional Moduli	Experimental [MPa]	Rule of Mixtures [MPa]
$\ell = 0.1$	E_1	137	318
	E_2	0.084	0.090
	G_{12}	0.075	0.029
$\ell = 0.2$	E_1	56	159
	E_2	0.070	0.077
	G_{12}	0.048	0.025
$\ell = 0.6$	E_1	22	53
	E_2	0.050	0.070
	G_{12}	0.042	0.023

Supplementary Table 1. Tabulation of collected initial moduli data for the various fiber spacings. Decreasing inter-fiber spacing leads to stiffer laminae. The rule of mixtures tends to overestimate the modulus compared to experimental values.

		Inter-Fiber Spacing mm		
		$l_1 = 0.1$	$l_2 = 0.2$	$l_3 = 0.6$
HGO Fitted Material Parameter	C_{10} $[MPa]$	0.0204	0.0119	0.006918
	k_1 $[MPa]$	24.3745	11.797	5.1578
	k_2	2.98e-8	2.1062e-8	2.1062e-8

Supplementary Table 2. Fitted Holzapfel-Gasser-Ogden (HGO) material parameters for the three different inter-fiber spacings. The values were determined through a non-linear least squares fit of the experimental data for the uniaxial extension of each sample along $\boldsymbol{\theta = 0°, 45°}$, and 90°.
